# Immunosuppressant Adherence Factors Differentiating Compliant and Non-Compliant Kidney Transplant Recipients

**DOI:** 10.3390/jcm12124081

**Published:** 2023-06-16

**Authors:** Piotr Ostrowski, Michał Kargul, Klaudia Gurazda, Anastasiia Skoryk, Marek Ostrowski, Marek Myślak, Jacek Różański, Edyta Skwirczyńska

**Affiliations:** 1 Department of General Surgery and Transplantation, Pomeranian Medical University, 70-111 Szczecin, Polandmostrowski@poczta.onet.pl (M.O.);; 2 Department of Nephrology and Kidney Transplantation, Provincial Integrated Hospital, Arkońska 4, 71-455 Szczecin, Poland; 3 Clinical Department of Nephrology, Transplantology and Internal Medicine, Pomeranian Medical University, 70-111 Szczecin, Poland

**Keywords:** kidney, transplant, adherence, immunosuppressant, drugs, factors

## Abstract

The purpose of this study is to find out the psychological factor characteristic of non-adherence patients. The study population comprised kidney transplant recipients aged between 18 and 82 years at least 3 months post-transplant who voluntarily agreed to answer a couple of fully anonymous questionnaires that questions pertaining to basic data, type of immunosuppressive drugs taken, and standardized questionnaires. Participants were recruited using direct routine, free-of-charge visits to specialist doctors in transplant clinics. There was no significant difference in the percentage of men and women in both adherence and non-adherence groups. Non-adherence patients were significantly younger compared to adherence patients. There was also a significant difference in the patient’s level of education. Adherence patients were better educated. No significant differences in criteria such as place of residence, having children or a partner, or way of living were observed. However, the emotion scale correlated negatively with the level of life orientation in both groups, but the level of the emotions scale and distractions subscale was negatively correlated with the level of self-esteem only for the adherence group. In future research, it would be worthwhile to focus on lifestyle and health-promoting behaviors in juxtaposition with the propensity for adherence.

## 1. Introduction

The maintenance of a functioning organ depends on many factors such as the condition of the donor and recipient, the duration of cold and warm ischemia, the surgical technique, and any surgical complications [1,2,3]. In addition, long-term postoperative care and the patient’s cooperation with the attending physician are also important [4].

Adherence to medication protocols and lifestyle regulations is an essential factor for successful kidney transplant outcomes [5]. Recent studies have sought to identify the differences between compliant and non-compliant patients. The findings demonstrate that both sociodemographic and psychosocial characteristics are associated with adherence behavior in kidney transplant recipients [6,7].

In our study, we have focused on the psychological aspect of the human factor in the form of the patient and the term “adherence”, and this consists of three main elements: compliance, persistence and continuity, and concordance. Adherence seems to be a complex behavior, and it is not necessarily directly associated with the psychological processing of organ transplantations [8]. The main aim of our study was to check the regularity of immunosuppressive drug use in patients after kidney transplantation, which, as the literature so far shows, is complicated [9,10,11,12,13,14]. If patients do not follow their doctor’s instructions and if they are not regular and disciplined in their medication, they may lose an organ, which has negative health consequences for the patient [15]. Moreover, even though non-adherence in a population of transplant recipients is costly on several levels (for instance, social, medical, and economic), it is resistant to change from a behavioral perspective [16]. Non-adherent patients were seven times more at risk of graft failure than adherent patients [17]. Unfortunately, this also undermines the substantial amount of work that medical staff has put in to ensure that patients can receive a new organ. Non-adherence is a major risk factor for rejection and allograft loss among transplant recipients [18,19,20]. In addition, such recipients generate very high economic costs. Non-adherent patients who reject transplants, due to their ignorance, are denying other people who would have followed medical advice the opportunity to receive an organ. We intended to develop a questionnaire tool to identify “non-adherence” patients. Methods for verifying answers that were honest and truthful needed to be determined.

## 2. Materials and Methods

The study population was kidney transplant recipients (*n* = 217) aged between 18 and 82 years at least 3 months post-transplant. Patients were invited to the study from two independent transplant clinics in the Szczecin area for a period of 2 years (January 2021–February 2023). Exclusion criteria included the following: rapidly progressive graft failure, severe neurocognitive disease, or inability to communicate fluently in Polish.

The questionnaire included basic data (gender, age, education level, the population of residence, having children, marital status, housing conditions, and occupation); type of immunosuppressive drugs taken; and standardized questionnaires such as the Coping Inventory for Stressful Situations (CISS), Rosenberg Self-Esteem Scale (SES), or Life Orientation Test-Revised (LOT-R). The mode of all questions was objective in order to make it as easy as possible for the subject to complete the questionnaire. In addition, we decided to add more questions to “mask” questions about the regular intake of immunosuppressive drugs. The masking questions were not subjected to statistical analysis—their purpose was to elicit more honest answers. The intention was to emphasize that the data were obtained from patients in the most objective way possible—patients were unaware that the study’s primary focus is on adherence. The questionnaire consisted of 118 questions in total (50 original questions and 68 in standardized tools). This anonymous survey provides valuable insight into the actual non-adherence rate, factors associated with non-adherence, and life situations that may complicate medication taking [16]. A particular part of the survey had to be completed by at least 90% in order to be statistically analyzed.

Participants were recruited by direct routine, free-of-charge visits to specialist doctors in transplant clinics. Patients were informed that participation in the study was voluntary, and the questionnaire was fully anonymous. Each patient who agreed to participate in the study received an A4 size questionnaire placed in an envelope. The participant was asked to complete the form in a separate room without the presence of other patients or staff, but if necessary (at the patient’s request), the staff dispelled doubts about the questions and explained their meaning. There was unlimited time to complete the questionnaire, and once completed, the patient was asked to drop the completed questionnaire into a special urn. After each day of the study, the researchers collected the surveys and systematically entered them into a spreadsheet (Excel 2019). After a dozen months of data collection, statistical analysis calculations were performed.

## 3. Results

To address the research questions, we carried out statistical analysis on the obtained database using IBM SPSS Statistics 25 software. We calculated basic descriptive statistics using the Kolmogorov–Smirnov test, Pearson’s correlation analysis, Student’s *t*-test for independent samples, Mann–Whitney U tests, χ^2^ tests, and Fisher’s exact tests. In this chapter, we used an alpha value threshold of 0.05 (*p* < 0.05).

Descriptive statistics are shown in Table 1. The Kolmogorov–Smirnov test showed that the distribution of results in the avoidance scale was similar to a normal distribution. In all other scales, the Kolmogorov–Smirnov test was statistically significant, which means that the distribution of said scales was significantly different from the normal distribution. However, both skewness and kurtosis were smaller than the absolute value of 2, so a parametrical test could be performed (George & Mallery, 2019).

### 3.1. Adherence and Demographic Aspects of Patients

In the following step, we performed several χ^2^ tests, Student’s *t*-test for independent samples, and Mann–Whitney *U* tests to find out if there are significant differences in demographic aspects between adherence and non-adherence patients. There was no significant difference in the percentage of men and women in both groups: χ^2^(1) = 0.34; *p* = 0.558 (Table 2).

There was a significant difference in the patient’s age: *t*(203) = −3.22; *p* = 0.001; *d* = 0.45. Non-adherence patients were significantly younger (M = 48.81; SD = 13.91) compared to adherence patients (M = 54.97; SD = 13.33).

There was also a significant difference in the patient’s level of education: *U* = 4625; *Z* = −2.59; *p* = 0.010; *r* = 0.18. Adherence patients were better educated, as observed in Table 3.

There were no significant differences in other analyzed variables (Table 4).

### 3.2. Behavioral Differences between Adherence and Non-Adherence Groups

In the following step, we performed several Fisher’s exact tests to find out if there are significant differences in behavioral aspects between adherence and non-adherence patients. There was one significant difference (Table 5). The adherence group declared eating less often at fixed, scheduled times: *Z* = −2.59; *p* = 0.010; *r* = 0.18. Adherence patients were better educated, as observed in Table 3. The size of the observed effect was weak. Interestingly, the difference in the regularity of meals was not statistically significant. There was also no difference in playing sports.

### 3.3. Stress Coping Styles in Adherence and Non-Adherence Groups

In the following step, we checked whether there is a significant difference in stress coping styles in adherence and non-adherence groups. A series of Student’s *t*-tests for independent samples were performed. However, no statistically significant results were found (Table 6).

### 3.4. Self-Esteem and Life Orientation in Adherence and Non-Adherence Groups

In the next step, we checked if there is a significant difference in the levels of self-esteem and life orientation. Student’s *t*-tests for independent samples were performed. However, no statistically significant results were recorded (Table 7).

### 3.5. Relationship between the Styles of Coping with Stress and the Self-Esteem

In the next step, whether there was a significant relationship between the level of styles of coping with stress and the self-esteem of the patients was checked. Therefore, a series of Pearson *r* correlation tests were performed separately for adherence and non-adherence groups. As observed in Table 8, there were two statistically significant relationships—both were in the adherence group. The level of the emotions scale and distractions subscale was negatively correlated with the level of self-esteem. The strength of the former correlation was moderately strong, while the latter was low. In the non-adherence group, both relationships were not statistically significant. Fisher’s *Z* test showed that the difference in the strength of the relationship in both groups between self-esteem and emotion scale was at the level of the statistical trend. No statistically significant results were found for the remaining relationships.

### 3.6. Relationship between the Styles of Coping with Stress and the Life Orientation of Patients

In the last step, we checked whether there was a significant relationship between coping with stress styles and the life orientation of patients. Therefore, a series of Pearson *r* correlation tests were performed separately for people with and without adherence. As shown in Table 9, there were seven statistically significant relationships. The task scale correlated positively with the life orientation index both in the adherence and non-adherence groups. The strength of the former correlation was low, and the latter was moderately high, although Fisher’s *Z* test did not show that the strength of the two compounds was statistically significantly different. The emotion scale correlated negatively with the level of life orientation in both adherence and non-adherence groups. In the adherence group, this relationship was strong, while in the non-adherence group, it was weak, and Fisher’s *Z* test showed that the difference in this strength was statistically significant. The avoidance scale positively correlated with the level of life orientation only in the non-adherence group. The relationship was weak. However, Fisher’s *Z* test showed that the difference in the strength (and sign) of this correlation in the adherence and non-adherence groups was statistically significant. The distraction subscale was negatively correlated with the level of life orientation only in the adherence group. The relationship was weak. Fisher’s *Z* test showed that the difference in the strength (and sign) of this correlation in the groups with and without adherence was statistically significant. The social diversion subscale correlated positively with the level of life orientation only in the non-adherence group. This relationship was moderately strong. Fisher’s *Z* test showed that the difference in this correlation’s strength (and sign) in the adherence and non-adherence groups was statistically significant. No statistically significant results were found for the remaining relationships.

## 4. Discussion

The purpose of our study was to find out the psychosocial factor characteristic of non-adherence patients. The age of the patient, the complexity of the immunosuppressive regimen, medication side effects, a lack of understanding of the recommendations, and a longer time period before transplantation are some of the most significant sociodemographic and medical factors that negatively affect adherence [21,22]. Other important sociodemographic and medical factors include psychosocial factors such as depression [23], anxiety [24], social functioning [25], and transplant-related stress [26]. Among the study group, nearly half of the patients were classified in the non-adherence group. Similarly to our study, Scheel et al. (2018) reported that non-adherence was associated with younger age of patients [9]. An interesting factor characterizing non-adherence patients in our study was the regularity of mealtimes. We found no other studies showing associations between the regularity of mealtimes and medication adherence. This area may be an interesting field for further exploration due to several psychological variables associated with eating habits such as depression, conscientiousness, and a sense of control. A better understanding of co-occurring variables may contribute to the easier identification of non-adherence patients, which will allow faster intervention in their case. Another interesting result was the comparison of stress coping styles and self-esteem in adherence and non-adherence patients. It was shown that in adherence patients using an emotion-based style and engaging in vicarious activities, these styles were negatively correlated with their self-esteem.

In addition, we found no studies that showed any consistent association between specific phases of stress and maladaptive non-adherence behavior in kidney transplant patients. Patients with higher levels of education have higher levels of discipline with respect to complying with medical recommendations. Other studies presented analogous results. Allograft loss was observed in those with lower education levels (P for trend = 0.03) by Elke S Schaeffner’s team [10]. Research from 2017 also presented that educational level resulted in non-adherence [11]. This may be due to their higher standard of living as well as a greater awareness of the negative consequences of noncompliance with medical recommendations. The Polish transplant care system has no interdisciplinary teams with a pharmacist in practice. The pharmacist would strictly explain the effects of drugs and other supplements that patients use, and the consequences of irregular use that negatively affect cellular metabolism are explained.

In their study, Breu-Dejean et al. (2016) showed that a single psychological intervention shortly after transplantation did not improve patients’ adherence translating into 10-year graft function [12]. Many psychological traits in humans are permanent. Even though the benefits of KT for QoL over treatments for end-stage renal illness are well recognized [27,28], KT patients continue to face several challenges following transplant [29,30,31]. A strict regimen of immunosuppressive medications and their associated side effects, frequent doctor visits and hospital stays, infections, and symptoms of anxiety and depression related to rejection episodes and the potential loss of the graft are some of the negative aspects of patients’ lives after KT [32,33,34,35]. The above results reflect psychological mechanisms regarding the constancy of certain psychological indicators [10]. In our study, as in the mentioned studies [15], we showed that both age and education level are determinants of patients’ adherence. At the same time, we showed that patients with a certain degree of regularity regarding their meals have a lower propensity for adherence. This may be due to the adopted care and broader awareness of healthy functioning and not only eating habits. Hence, as part of taking care of one’s health and body, there is also regularity in the use of immunosuppressive medications, which are an essential element of graft survival length after transplantation.

The questionnaire was designed in such a way that questions about the regularity of medication use were sandwiched between questions about lifestyle, food intake, sports participation, and other diseases. This design of the questionnaire was intended to lull the subjects’ alertness [8]. A limitation of our study was the fact that patients waited for follow-up appointments at the outpatient clinic, and our questionnaires were given to the clinic staff. It presumably did not escape the attention of some of them that there were several questions regarding the regularity of medication use despite the written information noting that the questionnaire was anonymous and would only be used for statistical purposes; patients may have been concerned that the doctor would inquire as to whether someone was or was not complying. Therefore, further research should think about introducing a form that could give comfort and a sense of security in order to allow completely honest answers. In further research, it would be worthwhile to focus on lifestyles and their health-promoting behaviors in juxtaposition with the propensity for adherence.

## Figures and Tables

**Table 1:** Descriptive statistics (Pearson’s correlation analysis).

	M	Me	SD	Sk.	Kurt.	Min.	Max.	D	*p*
Task	3.55	3.63	0.55	−0.60	0.85	1.80	4.75	0.07	0.013
Emotion	2.44	2.40	0.69	0.30	−0.12	1	4.53	0.06	0.037
Avoidance	2.90	2.88	0.55	0.15	0.10	1.50	4.56	0.04	0.200
Distraction	2.55	2.63	0.67	0.06	−0.04	1	4.38	0.07	0.022
Social Diversion	3.47	3.60	0.70	−0.23	0.06	1.20	5	0.09	<0.001
Self-esteem	2.32	2.40	0.30	−0.26	5.49	1	3.80	0.15	<0.001
Life orientation	3.04	3.10	0.50	−0.36	−0.28	1.70	4	0.10	<0.001
Age	52.33	54	13.88	−0.15	−0.89	20	82	0.08	0.003

**Table 2:** Adherence and gender.

		Adherence	
		Yes	No
Women	*N*	47	60
	%	51.60%	47.60%
Men	*N*	44	66
	%	48.40%	52.40%

**Table 3:** Adherence and education level.

		Adherence	
		Yes	No
basic	*N*	3	7
	%	3.30%	5.60%
professional	*N*	18	43
	%	19.80%	34.10%
secondary	*N*	44	53
	%	48.40%	41.30%
higher	*N*	26	23
	%	28.60%	18.30%

**Table 4:** Adherence and other demographic variables.

			Adherence		
			Yes	No	
Place of residence	<200.000	*N*	66	89	*U* = 5589; *Z* = −0.02; *p* = 0.983
		%	72.50%	72.40%	
	>200.000	*N*	25	34	
		%	27.50%	27.60%	
Kids	yes	*N*	62	94	χ^2^(1) = 1.08; *p* = 0.295
		%	68.10%	74.60%	
	no	*N*	29	32	
		%	31.90%	25.40%	
Partner	yes	*N*	74	99	χ^2^(1) = 0.90; *p* = 0.637
		%	81.30%	79.20%	
	no	*N*	17	26	
		%	18.70%	20.80%	
The way of living	alone	*N*	9	20	χ^2^(1) = 1.81; *p* = 0.178
		%	9.90%	16.30%	
	with family	*N*	82	103	
		%	90.10%	83.70%	

**Table 5:** Adherence to eating and playing sports by study subjects (Fisher’s test).

			Adherence		
			Yes	No	
Do you eat meals regularly?	Yes	*N*	65	94	*p* = 0.188
		%	72.20%	77.70%	
	Do not know	*N*	0	3	
		%	0.00%	2.50%	
	no	*N*	25	24	
		%	27.80%	19.80%	
I always eat at the same time	yes	*N*	38	75	*p* = 0.005 *V* = 0.22
		%	41.80%	64.10%	
	Do not know	*N*	5	3	
		%	5.50%	2.60%	
	no	*N*	48	39	
		%	52.70%	33.30%	
Do you play sports?	Yes	*N*	30	41	*p* = 0.486
		%	34.50%	37.60%	
	Do not know	*N*	1	4	
		%	1.10%	3.70%	
	no	*N*	56	64	
		%	64.40%	58.70%	

**Table 6:** Stress coping styles in adherence and non-adherence groups (Student’s test).

	Yes (*n* = 88)		No (*n* = 122)		*t*	*p*	95% CI		Cohen’s *d*
	M	SD	M	SD			LL	UL	
Task	3.61	0.52	3.51	0.56	1.21	0.229	−0.06	0.24	0.18
Emotion	2.48	0.71	2.40	0.67	0.84	0.402	−0.11	0.27	0.12
Avoidance	2.93	0.53	2.87	0.56	0.75	0.455	−0.09	0.21	0.11
Distraction	2.62	0.69	2.50	0.66	1.29	0.200	−0.06	0.31	0.18
Social Diversion	3.48	0.67	3.47	0.73	0.12	0.903	−0.18	0.21	0.01

**Table 7:** Self-esteem and life orientation in adherence and non-adherence groups.

	Yes (*n* = 88)		No (*n* = 122)		*t*	*p*	95% CI		Cohen’s *d*
	M	SD	M	SD			LL	UL	
Self-esteem	2.33	0.31	2.33	0.26	−0.08	0.939	−0.08	0.08	0.01
Life orientation	3.03	0.53	3.05	0.49	−0.17	0.864	−0.16	0.13	0.03

**Table 8:** Relationship between the styles of coping with stress and self-esteem.

		Self-Esteem		
		Adherence	Non-Adherence	Fisher’s *Z*
Task	*r*	−0.070	0.128	*Z* = −1.40
	*p*	0.518	0.165	*p* = 0.162
Emotion	*r*	−0.319	−0.056	*Z* = −1.93
	*p*	0.002	0.548	*p* = 0.054
Avoidance	*r*	−0.195	−0.062	*Z* = −0.95
	*p*	0.069	0.502	*p* = 0.342
Distraction	*r*	−0.238	−0.126	*Z* = −0.82
	*p*	0.025	0.172	*p* = 0.412
Social Diversion	*r*	−0.074	0.079	*Z* = −1.08
	*p*	0.494	0.395	*p* = 0.280

**Table 9:** Correlation of stress coping styles and life orientation of study subjects (Fisher’s test).

		Life Orientation		
		Adherence	Non-Adherence	Fisher’s *Z*
Task	*r*	0.235	0.365	*Z* = −1.01
	*p*	0.034	<0.001	*p* = 0.313
Emotion	*r*	−0.503	−0.254	*Z* = −2.07
	*p*	<0.001	0.008	*p* = 0.039
Avoidance	*r*	−0.123	0.205	*Z* = −2.33
	*p*	0.269	0.035	*p* = 0.020
Distraction	*r*	−0.222	0.063	*Z* = −2.03
	*p*	0.045	0.520	*p* = 0.042
Social Diversion	*r*	0.050	0.374	*Z* = −2.42
	*p*	0.657	<0.001	*p* = 0.016

## Data Availability

Not applicable.
